# Solvent-pumped evaporation concentration on paper in linear and radial geometries

**DOI:** 10.1063/5.0161199

**Published:** 2023-08-09

**Authors:** Richard R. A. Syms, Steven Wright

**Affiliations:** EEE Department, Imperial College London, Exhibition Road, London SW7 2AZ, United Kingdom

## Abstract

Solvent-pumped evaporation-driven concentration of an initial distribution of solutes on a porous substrate is considered in one and two dimensions. Approximate analytic solutions to the isotropic advection–dispersion equations are first found for a Gaussian kernel and an infinite domain, following the smoothed particle approximation. Analytic solutions for more general initial distributions are then found as sums of Gaussians, and comparison is made with numerical solutions. In each case, initial distributions are advected toward the stagnation point and concentrated. Two-dimensional analysis is then extended to describe anisotropy in permeability and diffusion, and hydrodynamic dispersion. Radial-flow experiments are performed using filter papers and water-soluble dyes. Diffusion coefficients, temperature and humidity profiles, and the evolution of spot distributions are measured. The results confirm minor anisotropy in permeability and diffusion, limited hydrodynamic dispersion, and largely uniform evaporation. Péclet numbers over 2500 are demonstrated. Evaporation-driven concentration provides a mechanism for solute transport over long timescales. Potential applications lie in the design of paper spray microanalytical devices operating by solvent pumping rather than capillary flow.

## I. INTRODUCTION

Evaporation-driven convection is a simple method of solute transport that requires no electrodes and can be used in low-cost microanalytic devices.^1,2^ Convection has been used to obtain concentration in microfluidic^3–6^ and paper^7–12^ devices, as well as crystallisation^13–16^ and self-assembly of microparticle arrays.^17–19^ Similar effects have been obtained by pervaporation through a porous layer^20–24^ and permeation into permeable substrates such as polydimethylsiloxane.^25–27^ Solvent evaporation can also provide a mechanism for self-assembly in drying films^28,29^ and chloride ion concentration in reinforced concrete.^30,31^

Transport is described using advection–dispersion equations (ADEs), partial differential equations that describe accumulation in the presence of advection and diffusion. In multi-component systems, they can also describe separation and hence form the basis of chromatography theory.^32^ In microfluidics, attention has mainly focused on one-dimensional (1D) systems, but 2D systems occur in radial chromatography^33^ and hydrology.^34–36^ However, work on paper microfluidics overwhelmingly involves capillary pumping and neglects rather than exploits evaporation-driven effects.

In a previous paper,^12^ we compared the performance of 1D and 2D evaporation-driven paper concentrators with infinite sources and highlighted the advantage of radial flow. Advective transport toward a single stagnation point provides an effective counter to diffusion. In devices of comparable Péclet numbers (which defines the relative significance of the two effects),^37^ radial flow offers faster concentration than linear flow after a longer filling time and may provide the basis for other 2D microfluidic devices. However, one difficulty preventing application may be the problem of finding analytic solutions for ADEs with varying coefficients, which masks potential capabilities.

Analytic solutions for special cases have been found in hydrology.^38–40^ More general initial value problems have been solved using power series^41^ and Laplace transforms,^36,42^ if necessary, using numerical inversion.^43^ As a last resort, ADEs can always be solved numerically.^44,45^ An alternative approach is offered by smoothed particle hydrodynamics (SPH), which provides approximate solutions in terms of assemblies of kernel functions. Originally developed in astrophysics,^46^ SPH has also been successful in fluid flow problems involving porous and anisotropic media.^47–49^ Here, we use an SPH approach to model solvent-pumped concentration of solute distributions in 1D and 2D porous systems, with the overall aim of understanding transport, concentration, and coalescence of an initial distribution of spots.

The paper is arranged as follows. In Sec. II, the ADEs for 1D and 2D geometries are reviewed. In Sec. III, SPH solutions are developed for Gaussian initial distributions in 1D domains and extended to general distributions. In Sec. IV, analysis is repeated for 2D domains. In Sec. V, the modifications needed to model anisotropy in permeability and diffusion are introduced. In Sec. VI, hydrodynamic dispersion is considered via numerical solutions. In Sec. VII, radial-flow experiments with dyes on filter papers are presented. Measurements of diffusion coefficients and temperature and humidity profiles are used to validate assumptions of isotropic diffusion and uniform evaporation. The evolution of initial distributions is then demonstrated, confirming anisotropic permeability but little hydrodynamic dispersion even with fan-assisted evaporation. Applications are considered in Sec. VIII, and conclusions are drawn in Sec. XI.

## II. ADVECTION–DISPERSION EQUATIONS

**FIG. 1.**
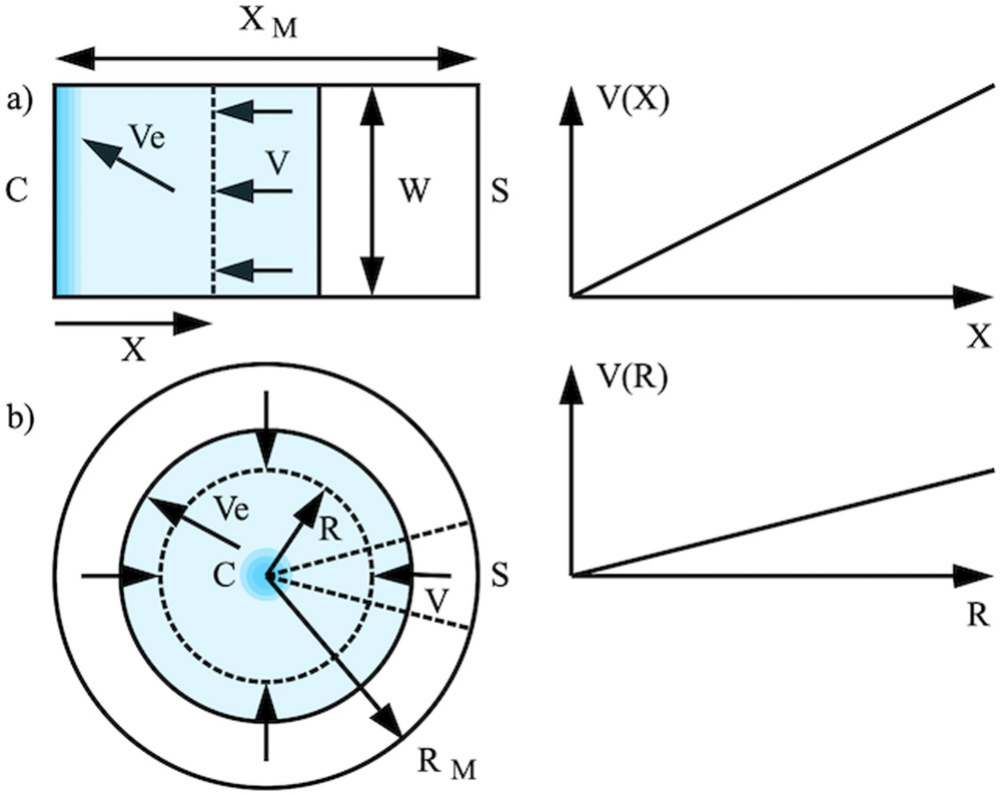
Arrangement for solvent-pumped concentration of solute in (a) linear and (b) radial geometries.

The ADEs for evaporation-concentration on a porous substrate have been derived elsewhere.^12^ Here, we give a brief outline. Figure 1 shows the geometries of (a) linear and (b) radial systems. In each case, a substrate of porosity ε, thickness *d*, and finite dimension $\left(X_{M}\;\mathrm{or}\;R_{M}\right)$ is assumed to contain an initial distribution of non-volatile solute, which is carried by a volatile solvent from a source *S* to a concentration point *C*, with solvent evaporation between. In the linear case, the source is distributed over a width *w*, and in the radial case, it is distributed round the perimeter. We assume that the substrates are horizontal (so gravity may be neglected) and that the capillary filling phase has ended (so that substrates are fully wetted). We also initially assume isotropy and uniform evaporation; these assumptions will be reviewed later.

For a 1D system, Darcy's law^50,51^ implies that the local solvent velocity *V* satisfies

$$V=-\frac{k}{\mu }\frac{dP}{dX}.$$ (1)

Here, *P* is the local fluid pressure, *k* is the permeability, and μ is the dynamic viscosity. Assuming a uniform evaporation flux $v_{e}$ per unit area from both sides together, continuity implies that the solvent flow velocity satisfies

$$\frac{dV}{dX}=-\frac{1}{\tau_{e}}.$$ (2)

Here, $\tau_{e}=d/v_{e}$ is a characteristic time constant. Combining the above, we obtain

$$\frac{k}{\mu }\frac{d^{2}P}{dX^{2}}=\frac{1}{\tau_{e}}.$$ (3)

Assuming V=0 at X=0 and $P=P_{atm}$ at $X=X_{M}$, where $P_{atm}$ is atmospheric pressure, we obtain

$$V=-\frac{X}{\tau_{e}},$$ (4)

$$P=P_{atm}-\frac{\mu (X_{M}^{2}-X^{2})}{2k\tau_{e}}.$$ (5)

The velocity, therefore, falls linearly toward the origin, while the pressure falls parabolically; it is this pressure drop that provides pumping.

The ADE can be derived by considering the balance of solute transport, diffusion, and accumulation. For a linear adsorption isotherm, the resulting equation can be written in terms of normalized quantities as

$$\frac{\partial C_{Lx}}{\partial t^{\prime }}=\frac{1}{Pe}\frac{\partial^{2}C_{Lx}}{\partial x^{2}}+x\frac{\partial C_{Lx}}{\partial x}+C_{Lx}.$$ (6)

Here, $C_{Lx}(x)$ is the liquid phase solute concentration, $x=X/X_{M}$ is the normalized distance, $Pe=X_{M}^{2}/D\tau_{e}$ is the Péclet number, and *D* is the diffusion coefficient, initially assumed to be both isotropic and constant. Finally, $t^{\prime }=R_{f}\;T/\tau_{e}$ is the normalized time, where *T* is the actual time and $R_{f}=1/\left(1+aF\right)$ is the retardation factor. Here, *a* is Henry’s coefficient and F=(1−ε)/ε is the volumetric ratio of the stationary and mobile phases. The left-hand side of the ADE describes accumulation, and the right-hand side models diffusion and convective transport.

For a radially symmetric 2D system, it is simple to show that the velocity profile and pressure distribution modify to $V=-R/2\tau_{e}$ and $P=\;P_{atm}-\mu \left(R_{M}^{2}-R^{2}\right)/4k\tau_{e}$, so the peak velocity and pressure drop are both halved compared to a 1D system with equivalent dimensions. For a radially symmetric concentration profile $C_{Lr}(r)$, the ADE can then be written in terms of normalized quantities as

$$\frac{\partial C_{Lr}}{\partial t^{\prime }}=\;\frac{1}{Pe}\left(\frac{1}{r}\right)\frac{\partial }{\partial r}\left(r\frac{\partial C_{Lr}}{\partial r}\right)+\left(\frac{r}{2}\right)\frac{\partial C_{Lr}}{\partial r}+C_{Lr}.$$ (7)

Here, the Péclet number is now $Pe=R_{M}^{2}/D\tau_{e}$. However, we are interested in the evolution of non-axisymmetric concentration profiles $C_{Lxy}(x,y)$ in the same radial flow field. In this case, the ADE can be written in Cartesian coordinates as

$$\begin{array}{r} \frac{\partial C_{Lxy}}{\partial t^{\prime }}=\frac{1}{Pe}\left\{\frac{\partial^{2}C_{Lxy}}{\partial x^{2}}+\frac{\partial^{2}C_{Lxy}}{\partial y^{2}}\right\}+\left\{\frac{x}{2}\frac{\partial C_{Lxy}}{\partial x}+\frac{y}{2}\;\frac{\partial C_{Lxy}}{\partial y}\right\}+C_{Lxy} \end{array}.$$ (8)

Questionable aspects above are the neglect of capillary transport, and the assumptions of uniform evaporation, isotropic permeability, and a constant, isotropic diffusion coefficient. Before proceeding, we make the following comments:

Capillary transport has been considered by many authors, both without^52–54^ and with^55–57^ evaporation. Its effect is to redistribute a solute deposited on a dry substrate. Once wetting is complete, evaporation will concentrate the new distribution by solvent pumping, and this step is the focus here. The evaporation rate will be determined by kinetics; again, this aspect has been considered by many authors.^58,59^ The main effect, convection, will be uniform if the paper temperature is uniform. Variations are likely to be small near the stagnation point, especially in the radial case. However, the evaporation rate will be different near the source, where heat transfer also takes place by conduction. This effect will be considered further in Sec. VII. Paper has preferred axes (the machine and cross directions). Anisotropic permeability leads to curved flow trajectories.^60,61^ Anisotropy may also alter the diffusion tensor.^62,63^ These effects will be considered further in Sec. V. Hydrodynamic dispersion in porous media leads to non-Fickian diffusion described by velocity-dependent axial and transverse diffusion coefficients.^49,64,65^ Because of the low flow velocity, these effects are likely to be limited here, but will be considered further in Sec. VI.

## III. 1D INITIAL VALUE PROBLEMS

We begin with initial value problems in one dimension. To do so, we first assume a trial solution to the ADE in the form of a moving Gaussian kernel, as

$$C_{Lx}=C_{L0}\sqrt{\frac{\;f_{x}}{\;f_{x0}}}\exp\left\{-f_{x}Pe\frac{{\left(x-x_{0}\right)}^{2}}{2}\right\}.$$ (9)

Here $x_{0}$ and $f_{x}$ are functions of $t^{\prime }$ that describe the time evolution of the center and width of the Gaussian, and $C_{L0}$ and $f_{x0}$ are constants. Differentiation, substitution into the ADE and equation of coefficients of terms ${\left(x-x_{0}\right)}^{n}$ separately then gives

$$\frac{dx_{0}}{dt^{\prime }}=-x_{0},$$ (10)

$$\frac{df_{x}}{dt^{\prime }}=2(\;f_{x}-f_{x}^{2}).$$ (11)

Both these equations may be solved by integration. For the initial conditions $x_{0}(0)=x_{00}$ and $f_{x}(0)=f_{x0}$, the results are

$$x_{0}(t^{\prime })=x_{00}\exp(-t^{\prime }),$$ (12)

$$f_{x}(t^{\prime })=\frac{\;f_{x0}e^{2t^{\prime }}}{\;f_{x0}(e^{2t^{\prime }}-1)+1}.$$ (13)

Because $x_{0}$ tends to zero, all initial distributions will evolve to a Gaussian at the origin. The solutions all tend from $f_{x0}$ at $t^{\prime }=0$ to unity as $t^{\prime }\to \infty$, and the steady state solution is

$$C_{Lx}=\frac{C_{L0}}{\sqrt{\;f_{x0}}}\exp\left(-Pe\frac{x^{2}}{2}\right).$$ (14)

The solutions above are exact for infinite space, but do not satisfy the boundary conditions for a finite concentrator. To obtain a suitable solution, we assume mirrored initial conditions. In this case, the analytic solution becomes

$$C_{Lx}=C_{L0}\sqrt{\frac{\;f_{x}}{\;f_{0}}}[\exp\left\{-f_{x}Pe\frac{{\left(x-x_{0}\right)}^{2}}{2}\right\}+\exp\left\{-f_{x}Pe\frac{{\left(x+x_{0}\right)}^{2}}{2}\right\}].$$ (15)

By symmetry, the solution derivative must now be zero at x=0. If the Gaussian terms always decay rapidly enough, the modified solution can be a good approximation for a finite concentrator over the reduced domain 0≤x≤1.

**FIG. 2.**
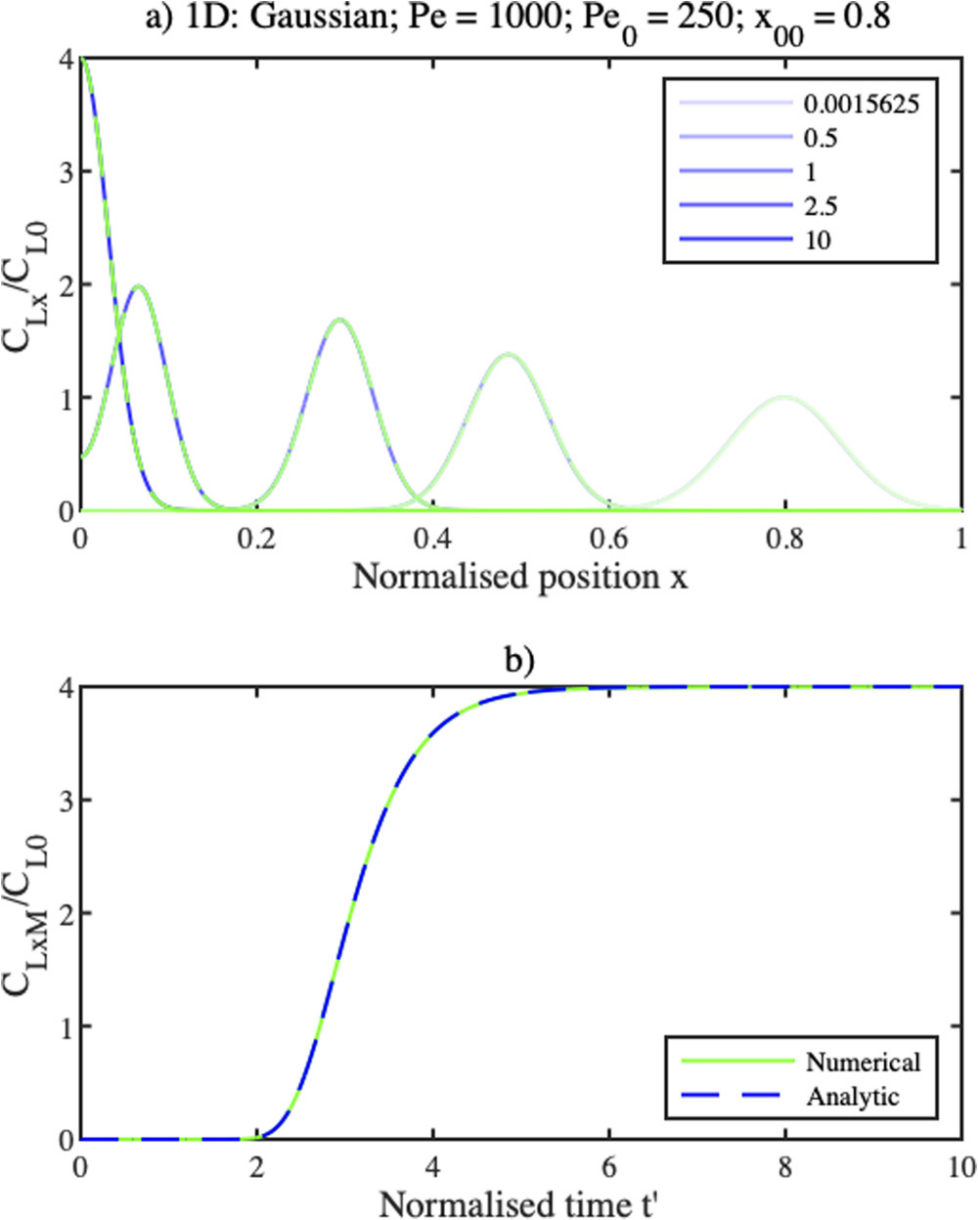
(a) Concentration profiles $C_{Lx}$ at different times $t^{\prime }$, for a Gaussian initial profile, (b) concentration variation at x=0.

Figure 2(a) compares the analytic solution (blue), with a numerical solution of the ADE (green), for Pe=1000 and an initial Gaussian distribution with a half-width and position defined by $Pe_{0}=f_{x0}Pe=250\;\mathrm{and}\;x_{0}=0.5$. The solute profile initially travels to the left, narrowing and increasing in height as it does. When it reaches the origin, it is trapped, and gradually evolves into a half-Gaussian. Figure 2(b) shows a similar comparison for the time variation of the concentration at x=0. Its value is initially zero, when the Gaussian is some distance from the origin. As it approaches the origin, it rises rapidly and then tends to a steady state. In each case, there is excellent agreement between the analytic and numerical solutions.

Because the ADE is linear, solutions for arbitrary initial distributions can be constructed as sums of Gaussians. Unfortunately, the expansion coefficients cannot be determined uniquely. However, reasonable approximations are possible if care is taken to manage the overlap of adjacent terms. Good results can be obtained using sums of identical, offset Gaussians, as

$$C_{Lx}=\sqrt{\frac{\;f_{x}}{\;f_{0}}}\sum_{i=1}^{N+1}a_{i}\left[\exp\left\{-f_{x}Pe\frac{{\left(x-x_{i}\right)}^{2}}{2}\right\}+\exp\left\{-f_{x}Pe\frac{{\left(x+x_{i}\right)}^{2}}{2}\right\}\right],$$ (16)

with

$$x_{i}(t^{\prime })=x_{0i}\exp(-t^{\prime })\;f_{x}(t^{\prime })=\frac{\;f_{x0}e^{2t^{\prime }}}{\;f_{x0}(e^{2t^{\prime }}-1)+1}.$$ (17)

Here, the terms $x_{i}$ are a set of N+1 centers with separation Δ=1/N, and the terms $a_{i}$ are expansion coefficients chosen to match the initial conditions, as

$$\begin{array}{rl} & x_{i}=0,\;\;a_{i}=C_{L0}(x_{i})/2K,\\ & x_{i}\neq 0,\;\;a_{i}=C_{L0}(x_{i})/K. \end{array}$$ (18)

The factor of 2 is used to prevent double counting at $x_{i}=0$ and the weighting factor *K* is introduced to compensate for the overlap of adjacent Gaussian terms, as

$$\begin{array}{r} K=1+2\left\{\;\exp\;\left[\;-\frac{Pe_{0}\Delta^{2}}{2}\right]+\exp\;\left[\;-\frac{Pe_{0}{\left(2\Delta \right)}^{2}}{2}\right]+\exp\;\left[\;-\frac{Pe_{0}{\left(3\Delta \right)}^{2}}{2}\right]\cdots \;\right\}. \end{array}$$ (19)

A smooth approximation may be obtained by assuming a suitable value for the initial Péclet number $Pe_{0}=f_{x0}Pe$ of each Gaussian. If $Pe_{0}$ is too small, and the centers are too widely spaced, the approximate distribution will contain ripples. However, if $Pe_{0}$ is too large, it will be difficult to model fast-varying distributions. Good results are obtained with $Pe_{0}=2N^{2}$. In this case, $Pe_{0}\Delta^{2}/2=1$, so that

$$K=1+2\left\{e^{-1}+e^{-4}+e^{-9}+\cdots \right\}\approx \sqrt{\pi }.$$ (20)

**FIG. 3.**
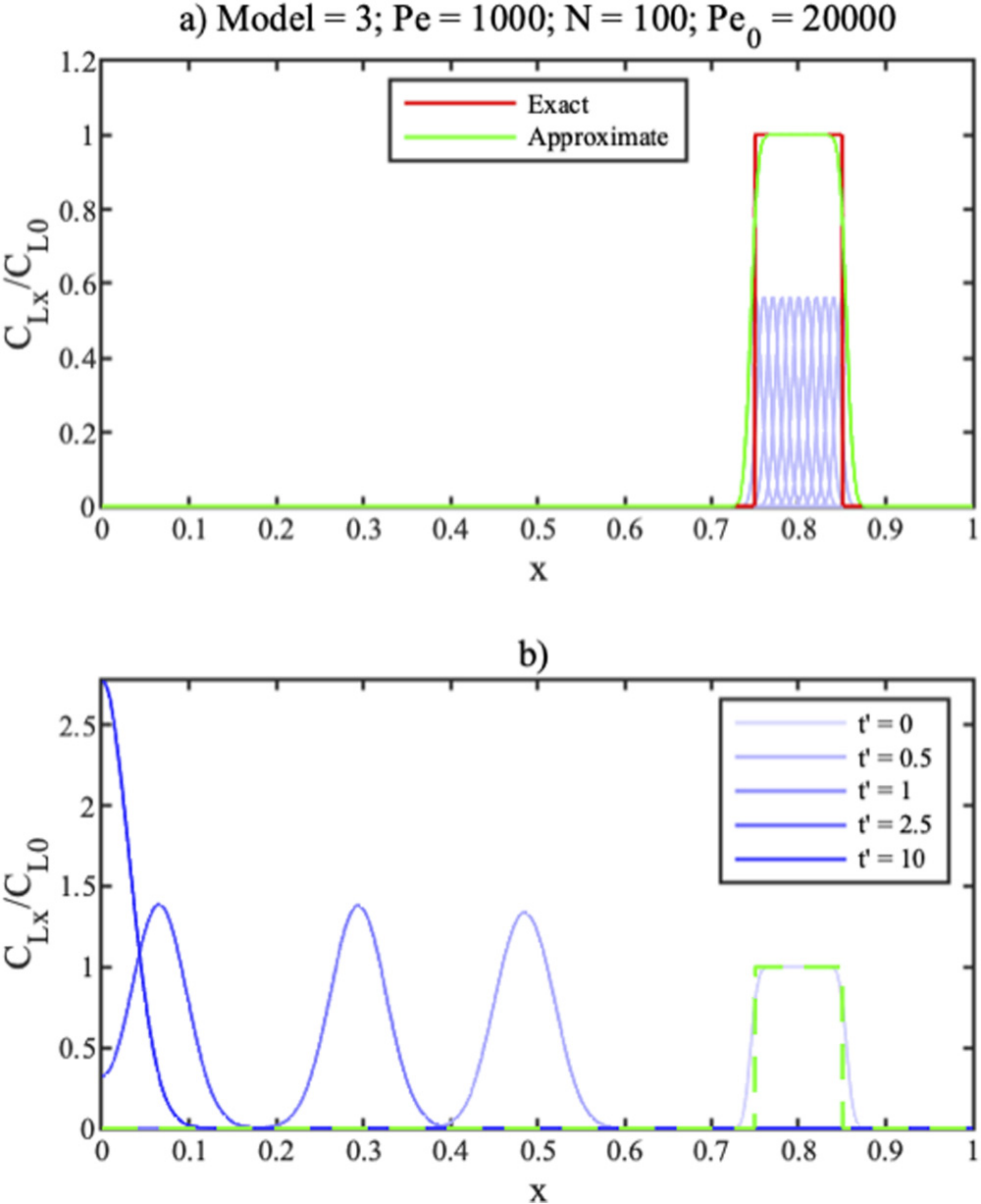
(a) Expansion and (b) concentration profiles at different times for Pe=1000 for a rectangular initial distribution.

Figure 3(a) shows such an expansion for a rectangular distribution from x=0.75 to x=0.85, with Pe=1000.

For this case, N=100 and $Pe_{0}=20000$. Individual Gaussians are shown in light blue, their summation in green and the target distribution in red. Only a small number of Gaussians can be seen because the remainder has zero amplitude. Adjacent terms overlap, and their amplitudes lie some way below the target profile because of the factor *K*. However, the overall sum yields a realistic approximation to the target profile. Its flat top is recreated smoothly; its edges are modeled less abruptly but become sharper if *N* is increased. Figure 3(b) shows the predicted concentration profile at the different times $t^{\prime }$ in the legend (blue); these agree almost exactly with a finite difference solution of the ADE for the same initial distribution (green).

## IV. 2D INITIAL VALUE PROBLEMS

We now repeat the process for 2D initial value problems. We first assume a trial solution to the ADE for a single offset Gaussian kernel as

$$C_{Lxy}=C_{L0}\sqrt{\frac{\;f_{x}f_{y}}{\;f_{x0}f_{y0}}}\exp\left\{-Pe\frac{\;f_{x}{\left(x-x_{0}\right)}^{2}+f_{y}{\left(y-y_{0}\right)}^{2}}{4}\right\}.$$ (21)

Here $x_{0}$, $y_{0}$, $f_{x}$, and $f_{y}$ are all functions of $t^{\prime }$ and describe the evolution of the Gaussian center and shape, and $C_{L0}$, $f_{x0}$, and $f_{y0}$ are constants. For the initial conditions $f_{x}(0)=f_{x0}$, $f_{y}(0)=f_{y0}$, $x_{0}(0)=x_{00}$, and $y_{0}(0)=y_{00}$, the solutions are

$$x_{0}=x_{00}\exp(-t^{\prime }/2),\;\;f_{x}=\frac{\;f_{x0}e^{t^{\prime }}}{\;f_{x0}(e^{t^{\prime }}-1)+1},$$ (22)

$$y_{0}=y_{00}\exp(-t^{\prime }/2),\;\;\;f_{y}=\;f_{y}=\frac{\;f_{y0}e^{t^{\prime }}}{\;f_{y0}(e^{t^{\prime }}-1)+1}.$$ (23)

These solutions are analogous to previous 1D results; but time constants have been doubled by the halved flow velocity. Once again, the solutions describe the evolution of an initial distribution to a steady state at the stagnation point, but no extra solution components are required to satisfy boundary conditions. Provided the Gaussian is not too extended, the solution is again a good approximation over the domain 0≤r≤1.

**FIG. 4.**
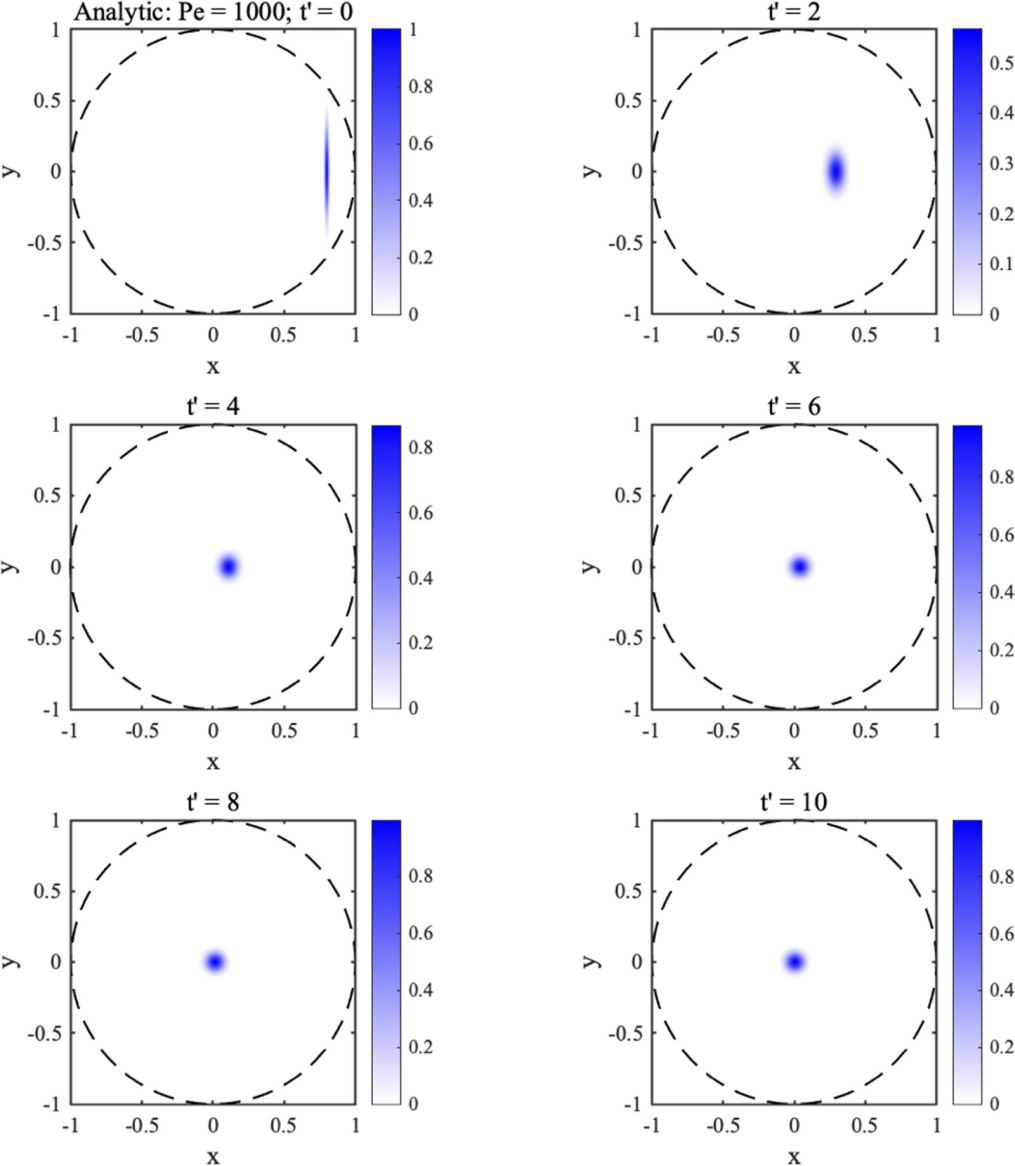
Concentration profiles calculated using the analytic solution at different times $t^{\prime }$, for Pe=1000, and an offset elliptical Gaussian initial distribution.

The left-hand equations imply that motion is always toward the origin. Similarly, the right-hand equations imply that the spot shape gradually evolves, with the limiting values $f_{x\infty }=f_{y\infty }=1$ showing that the eventual shape is circular. For example, Fig. 4 shows concentration profiles calculated using the analytic solution at the different times $t^{\prime }$ in the legend, for Pe=1000, and an offset elliptical Gaussian initial distribution with $Pe_{0x}=20Pe,\;Pe_{0y}=Pe/20$, $x_{0}=0.8$ and $y_{0}=0$. As $t^{\prime }$ increases, the distribution gradually tends to the origin and becomes more and more circular. We have verified that there is excellent agreement with numerical solution of the ADE for the same initial distribution. It is simple to show that the steady state solution is

$$C_{Lxy}=\frac{C_{L0}}{\;f_{0}}\exp\left(-Pe\frac{x^{2}+y^{2}}{4}\right).$$ (24)

Comparison with Eq. (14) shows that the peak width is doubled in the 2D case.

The procedure above may easily be adapted to model the evolution of a set of Gaussian spots with different sizes, shapes, and centers, using one term for each spot. A different approach is again required for arbitrary distributions. In this case, a solution may be constructed as a sum of displaced circular Gaussians, by assuming $f_{x}=f_{y}=f_{r}$ and $f_{x0}=f_{y0}=f_{r0}$, and writing

$$C_{Lxy}=\frac{\;f_{r}}{\;f_{r0}}\sum_{i=1}^{N+1}\sum_{\;j=1}^{N+1}a_{ij}e^{\left(-f_{r}Pe\frac{{\left(x-x_{ij}\right)}^{2}+{\left(y-y_{ij}\right)}^{2}}{4}\right)},$$ (25)

with

$$x_{ij}(t^{\prime })=x_{0ij}e^{-\frac{t^{\prime }}{2}},\;\;y_{ij}(t^{\prime })=y_{0ij}e^{-\frac{t^{\prime }}{2}},\;\;f_{r}(t^{\prime })=\frac{\;f_{r0}e^{t^{\prime }}}{\;f_{r0}(e^{t^{\prime }}-1)+1}.$$ (26)

Here, the coordinates $x_{ij}$ and $y_{ij}$ are a grid of centers with separation Δ=1/N, and the terms $a_{ij}$ are coefficients chosen to match the initial condition $C_{L0}(x,y)$ as

$$a_{ij}=C_{0}\left(x_{ij},x_{ij,}\right)/K.$$ (27)

A smooth approximation may again be obtained by assuming a suitable value for the initial Péclet number of each Gaussian. We have obtained good results with $Pe_{0}=4N^{2}$. Numerical summation of the Gaussians contributing to each point then shows that

K≈π. (28)

**FIG. 5.**
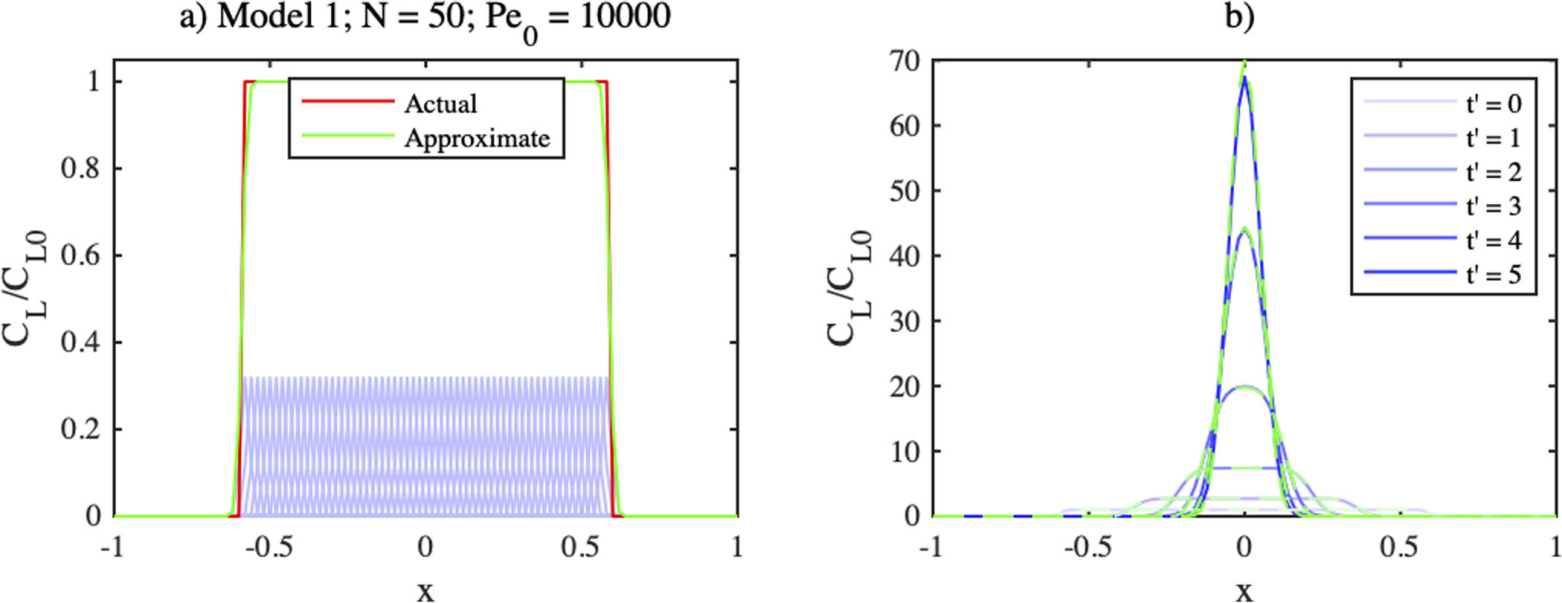
Line-scans in the *x* direction of (a) expansion and (b) concentration profiles at different times, for Pe=1000, a disk-shaped initial distribution up to r=0.6 and N=50.

Figure 5(a) shows line-scans in the *x*-direction of an expansion for a disk-shaped distribution extending up to r=0.6, with Pe=1000. Here, we choose N=50 and $Pe_{0}=10000$. Individual Gaussians are shown in blue, their sum in green, and the target distribution in red. Once again, adjacent Gaussians overlap, and their amplitudes lie below the target profile because of the factor *K*. However, the sum yields a realistic approximation. Figure 5(b) shows the predicted concentration profiles at the different times $t^{\prime }$ in the legend (blue); these agree almost exactly with a finite difference solution of the ADE for the same initial distribution (green). The corresponding *y*-variations are identical.

**FIG. 6.**
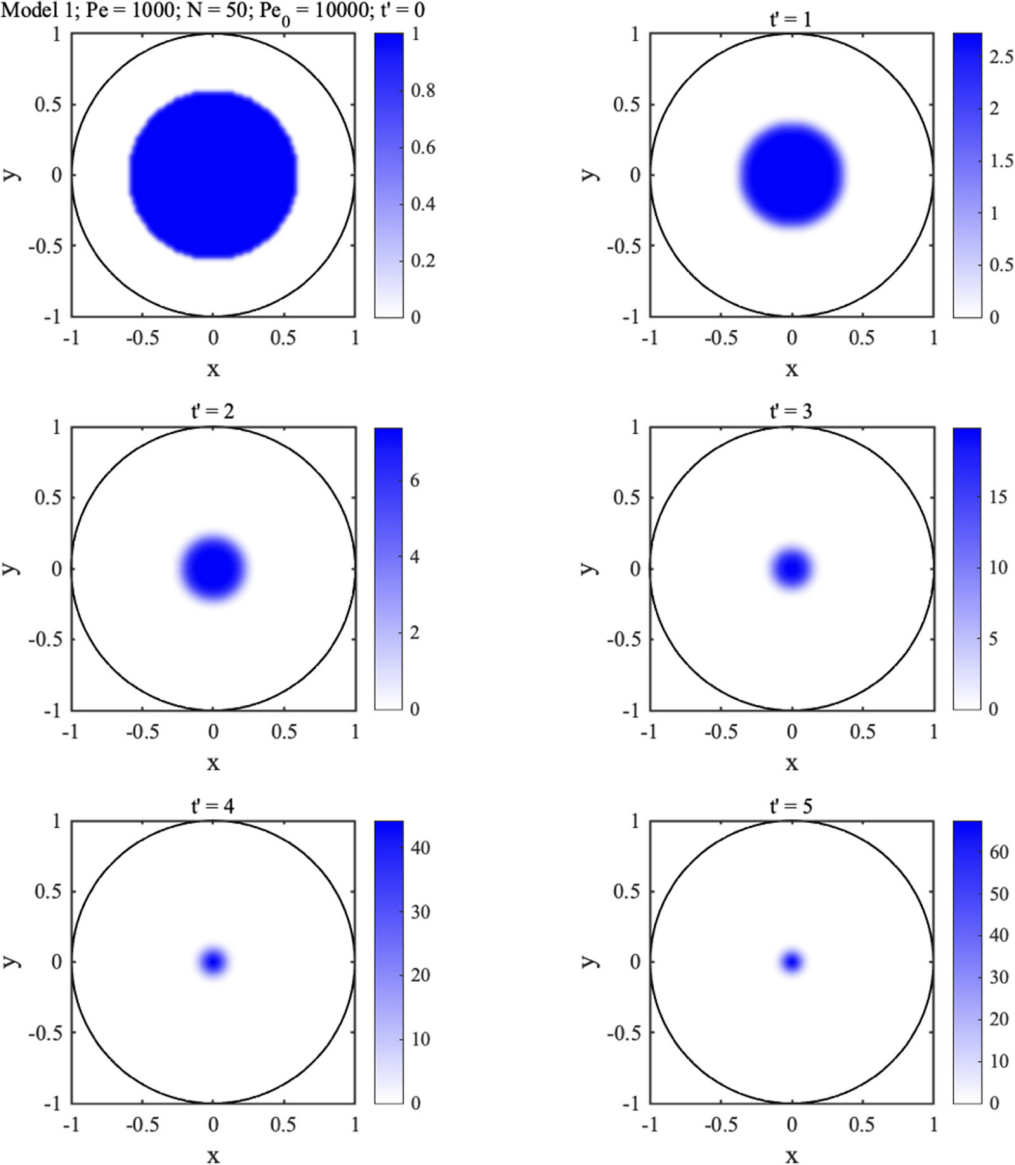
Concentration profiles calculated using the analytic solution at the different times $t^{\prime }$ indicated, for Pe=1000, and a disk-shaped initial distribution.

Figure 6 shows 2D plots of the concentration profile at different times $t^{\prime }$, which clearly evolves from a uniform disk to a centered Gaussian peak.

## V. ANISOTROPY

We now revisit the assumption of isotropy. If the Cartesian axes are aligned with the principal directions of an anisotropic substrate, solvent flow may be described by velocities $V_{x}(X)$ and $V_{y}(Y)$. Continuity implies that

$$\frac{\partial V_{x}}{\partial X}+\frac{\partial V_{y}}{\partial Y}=-\frac{1}{\tau_{e}}.$$ (29)

In the same way, Darcy's law may be taken for an anisotropic substrate as^60,61^

$$V_{x}=-\frac{k_{x}}{\mu }\frac{\partial P}{\partial X},\;\;V_{y}=-\frac{k_{y}}{\mu }\frac{\partial P}{\partial Y}.$$ (30)

Here, $k_{x}$ and $k_{y}$ are the permeabilities in the two directions. Combining the above we obtain

$$\frac{k_{x}}{\mu }\frac{\partial^{2}P}{\partial X^{2}}+\frac{k_{y}}{\mu }\frac{\partial^{2}P}{\partial Y^{2}}=\frac{1}{\tau_{e}}.$$ (31)

A symmetric solution for the pressure with stagnation at R=0 and $P=P_{atm}$ at $R=R_{M}$ can be found as

$$P(R)=\;P_{atm}-\frac{\mu (R_{M}^{2}-R^{2})}{2(k_{x}+k_{y})\tau_{e}}.$$ (32)

This result again implies that the pressure falls parabolically toward the origin, but that the velocity components are now different,

$$V_{x}=-\frac{k_{x}X}{(k_{x}+k_{y})\tau_{e}},\;\;\;V_{y}=-\frac{k_{y}Y}{(k_{x}+k_{y})\tau_{e}}.$$ (33)

We can write these alternatively as

$$V_{x}=-\frac{X}{\tau_{e}}\left(\frac{1+\alpha }{2}\right),\;\;\;V_{y}=-\frac{Y}{\tau_{e}}\left(\frac{1-\alpha }{2}\right).$$ (34)

Here, α is a coefficient that describes the flow anisotropy, namely,

$$\alpha =\frac{k_{x}-k_{y}}{k_{x}+k_{y}}.$$ (35)

Clearly, α=0 represents isotropy. Macroscopic solvent streamlines then have the form

$$\frac{dY}{dX}=\frac{Y}{X}\frac{\left(1-\alpha \right)}{\left(1+\alpha \right)}.$$ (36)

Integrating from $\left(X_{0},Y_{0}\right)$, we then have

$$\frac{Y}{Y_{0}}={\left(\frac{X}{X_{0}}\right)}^{\frac{\left(1-\alpha \right)}{\left(1+\alpha \right)}}.$$ (37)

For isotropic permeability (α=0), all trajectories are straight; for anisotropic permeability, oblique trajectories are curved. This observation allows estimation of anisotropy in permeability from spot trajectories.

For anisotropic media, we may also assume that diffusion is described by different diffusion coefficients $D_{x}$ and $D_{y}$,^62,63^ written conveniently as $D_{x}=\beta D$ and $D_{y}=\beta^{-1}D$, where $D=\sqrt{D_{x}D_{y}}$ and β=1 represents isotropy. The ADE can again be derived by considering the balance of solute transport, diffusion, and accumulation, as

$$\begin{array}{rl} \frac{\partial C_{L}}{\partial t^{\prime }} & =\frac{1}{Pe}\left\{\beta \frac{\partial^{2}C_{L}}{\partial x^{2}}+\beta^{-1}\frac{\partial^{2}C_{L}}{\partial y^{2}}\right\}\\ & \;+\left\{\frac{(1+\alpha )x}{2}\frac{\partial C_{L}}{\partial x}+\frac{(1-\alpha )y}{2}\;\frac{\partial C_{L}}{\partial y}\right\}+C_{L}. \end{array}$$ (38)

A solution for a single offset elliptical Gaussian spot may again be attempted in the form of Eq. (21), which for convenience we repeat here,

$$C_{Lxy}=C_{L0}\sqrt{\frac{\;f_{x}f_{y}}{\;f_{x0}f_{y0}}}\exp\left\{-Pe\frac{\;f_{x}{\left(x-x_{0}\right)}^{2}+f_{y}{\left(y-y_{0}\right)}^{2}}{4}\right\}.$$ (39)

Here $C_{L0}$, $f_{x0}$, and $f_{y0}$ are again constants, and the functions $x_{0}$, $y_{0}$, $f_{x}$, and $f_{y}$ now satisfy

$$\frac{dx_{0}}{dt^{\prime }}=-\frac{x_{0}}{2}(1+\alpha ),\;\;\frac{df_{x}}{dt^{\prime }}=f_{x}(1+\alpha )-\beta f_{x}^{2},$$ (40)

$$\frac{dy_{0}}{dt^{\prime }}=-\frac{y_{0}}{2}(1-\alpha ),\;\;\frac{df_{y}}{dt^{\prime }}=f_{y}(1-\alpha )-\beta^{-1}f_{y}^{2}.$$ (41)

For the initial conditions $x_{0}(0)=x_{00}$, $y_{0}(0)=y_{00}$, $f_{x}(0)=f_{x0}$, and $f_{y}(0)=f_{y0}$, the solutions are

$$x_{0}=x_{00}e^{-\frac{t^{\prime }(1+\alpha )}{2}},\;\;f_{x}=\frac{\;f_{x0}e^{t^{\prime }}}{\gamma f_{x0}(e^{t^{\prime }}-1)+1},$$ (42)

$$y_{0}=y_{00}ee^{-\frac{t^{\prime }(1-\alpha )}{2}},\;\;f_{y}=\frac{\;f_{0y}e^{t^{\prime }}}{\delta f_{y0}(e^{t^{\prime }}-1)+1}.$$ (43)

Here, the constants γ and δ are given by

$$\gamma =\frac{\beta }{\left(1+\alpha \right)},\;\;\delta =\frac{1}{\beta (1-\alpha )}.$$ (44)

We now consider the implications, starting with peak position. The center of the Gaussian spot again tends to the origin, and the time variations of $x_{0}$ and $y_{0}$ are simply different exponentials. However, the trajectory slope is now

$$\frac{dy_{0}}{dx_{0}}=\frac{y_{0}}{x_{0}}\frac{\left(1-\alpha \right)}{\left(1+\alpha \right)}.$$ (45)

Solute spot trajectories therefore follow solvent streamlines. We now consider the peak shape. In each case, the solution rises from the starting value to a limiting steady state, given by

$$f_{x\infty }=\frac{1}{\gamma }=\frac{\left(1+\alpha \right)}{\beta },\;\;f_{y\infty }=\frac{1}{\delta }=\beta (1-\alpha ).$$ (46)

If β=1, results for $f_{y}$ are obtained by reversing the sign of α in results for $f_{x}$. Generally, the final spot shape depends on both α and β. For example, $f_{x\infty }$ is increased (and the corresponding Gaussian width is decreased) if β<1 (so that $D_{x}$ is relatively low) or if α>1 (so that $V_{x}$ is high). However, an initially circular spot will remain so despite anisotropy if γ=δ, when differences in diffusion are exactly balanced by differences in advection. This requires the anisotropic coefficients to be related by

$$\alpha =\frac{\beta^{2}-1}{\beta^{2}+1},\;\;\beta =\sqrt{\frac{1+\alpha }{1-\alpha }}.$$ (47)

**FIG. 7.**
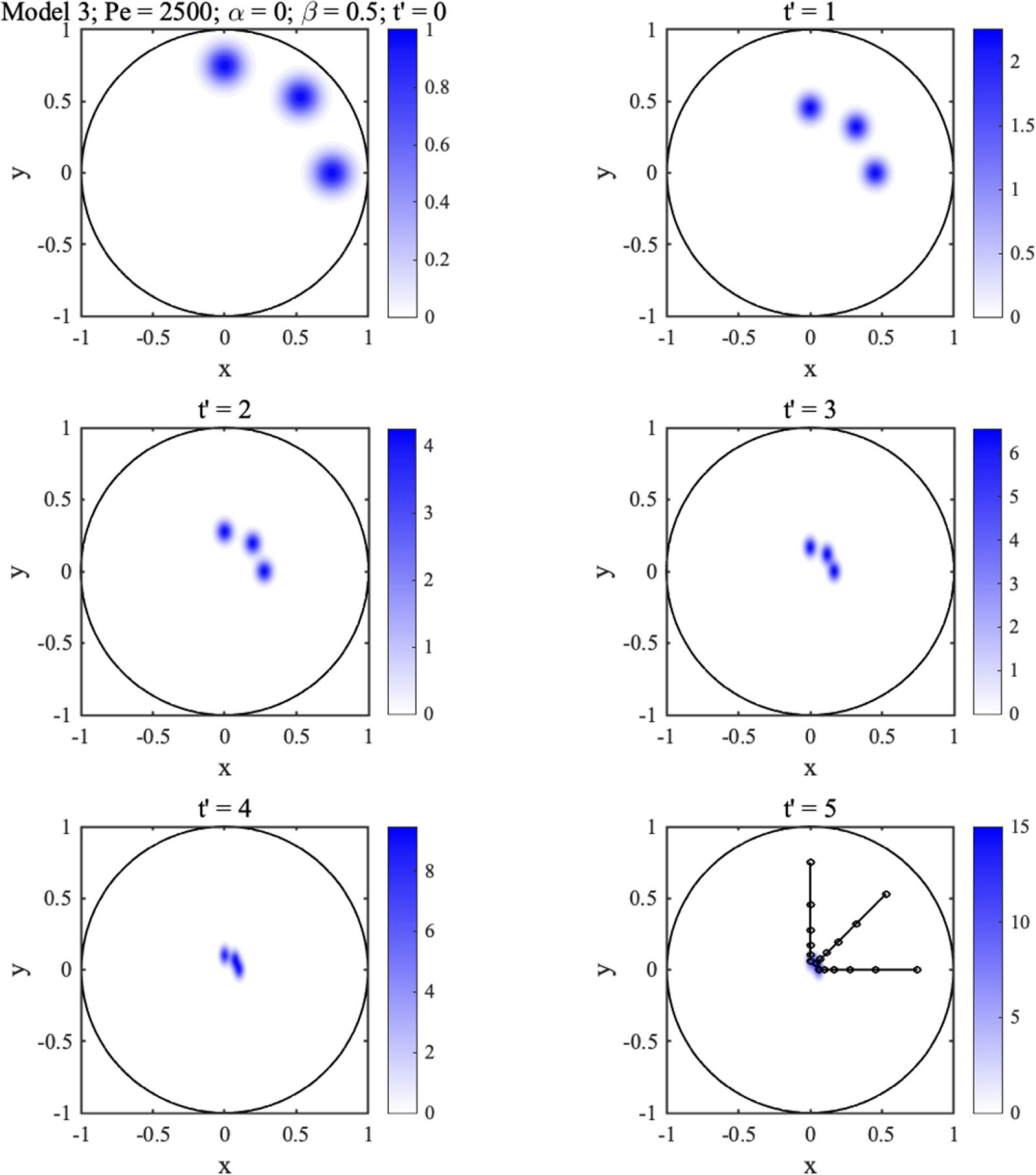
2D concentration distributions at different times for circular Gaussian spots with Pe=2500, α=0,β=0.5.

**FIG. 8.**
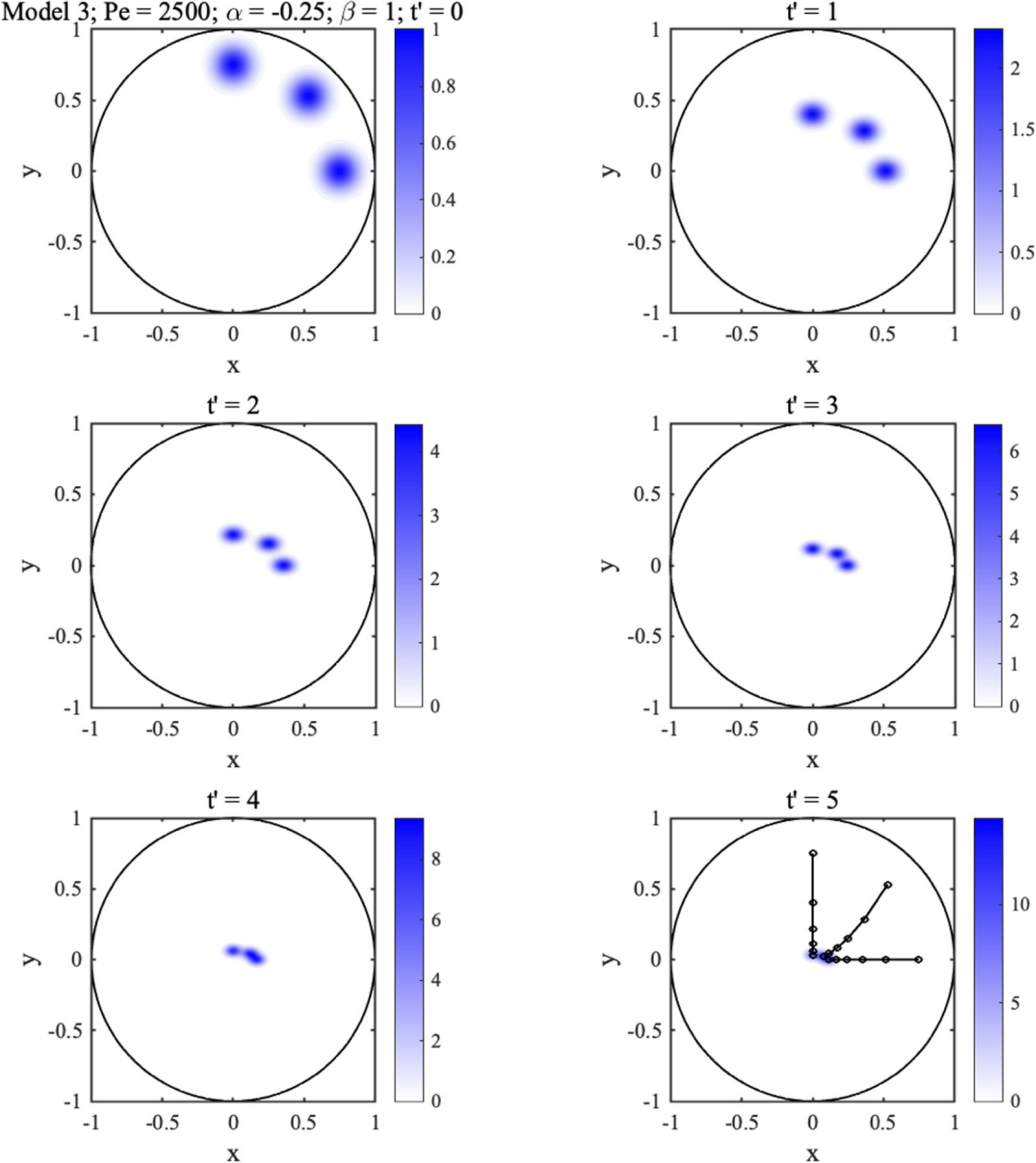
2D concentration distributions at different times for circular Gaussian spots with Pe=2500, α=−0.25,β=1.

Figures 7 and 8 show 2D concentration distributions at different normalized times $t^{\prime }$ for three initially circular spots with $Pe_{0}=250$, assuming that Pe=2500. The superimposed black lines show spot trajectories. In each case, the three spots are gradually transported toward the center, concentrating as they move. In Fig. 7, α=0,β=0.5, so permeability is isotropic, and all trajectories are straight. However, diffusion is anisotropic, so the spots gradually become elliptical with their long axes in the *y*-direction.

In Fig. 8, α=−0.25,β=1 so diffusion is isotropic, but permeability is anisotropic. Oblique trajectories now become curved and spots elliptical with their long axes in the *x*-direction. These results imply that diffusion anisotropy can be estimated from spot shapes if isotropy in permeability is already known.

## VI. HYDRODYNAMIC DISPERSION

We now briefly consider the possibility of hydrodynamic dispersion, assuming anisotropic permeability but for simplicity neglecting diffusion anisotropy. In this case, the components of the diffusion tensor may be written as^49^

$$\begin{array}{cc} & D_{xx}=D+\alpha_{T}|V|+(\alpha_{L}-\alpha_{T})\frac{V_{x}^{2}}{\left|V\right|},\\ & D_{xy}=D_{yx}=(\alpha_{L}-\alpha_{T})\frac{V_{x}V_{y}}{\left|V\right|},\\ & D_{yy}=D+\alpha_{T}|V|+(\alpha_{L}-\alpha_{T})\frac{V_{y}^{2}}{\left|V\right|}. \end{array}$$ (48)

Here, $\alpha_{L}$ and $\alpha_{T}$ are coefficients of longitudinal and transverse dispersion, and $V=V_{x}i+V_{y}j$ is the velocity vector. We can also define the normalized quantities,

$$\begin{array}{cc} & d_{xx}=\frac{D_{xx}}{D}=\left\{1+\beta_{T}|v|+(\beta_{L}-\beta_{T})\frac{v_{x}^{2}}{\left|v\right|}\right\},\\ & d_{xy}=\frac{D_{xy}}{D}=d_{yx}=\frac{D_{yx}}{D}=(\beta_{L}-\beta_{T})\frac{v_{x}v_{y}}{\left|v\right|},\\ & d_{yy}=\frac{D_{yy}}{D}=\left\{1+\beta_{T}|v|+(\beta_{L}-\beta_{T})\frac{v_{y}^{2}}{\left|v\right|}\right\}. \end{array}$$ (49)

Here, $\beta_{L,T}=\frac{\alpha_{L,T}|V|}{D|v|}$ and $v=v_{x}i+v_{y}j$ is a velocity vector normalized to the maximum. The ADE may then be written as

$$\begin{array}{rl} \frac{\partial C_{L}}{\partial t^{\prime }} & =\frac{1}{Pe}\left\{\begin{array}{c} \frac{\partial }{\partial x}\left(d_{xx}\frac{\partial C_{L}}{\partial x}+d_{xy}\frac{\partial C_{L}}{\partial y}\right)\\ +\;\frac{\partial }{\partial y}\left(d_{xy}\frac{\partial C_{L}}{\partial x}+d_{yy}\frac{\partial C_{L}}{\partial y}\right) \end{array}\right\}\\ & \;+\left\{\frac{(1+\alpha )x}{2}\frac{\partial C_{L}}{\partial x}+\;\frac{(1-\alpha )y}{2}\frac{\partial C_{L}}{\partial y}\right\}+C_{L}. \end{array}$$ (50)

**FIG. 9.**
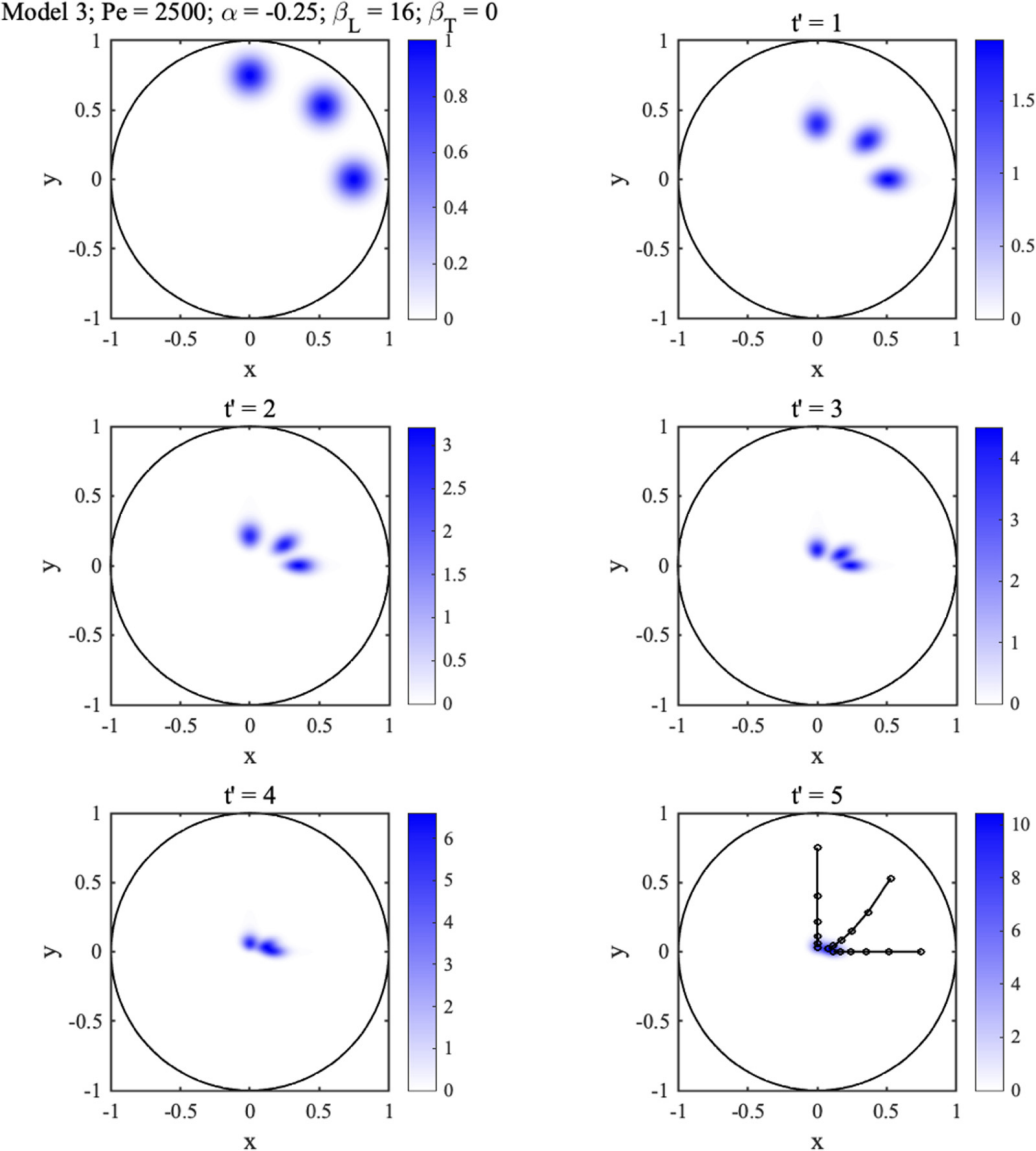
2D concentration distributions at different times for initially circular Gaussian spots with Pe=2500, α=−0.25, $\beta_{L}=\;16$, $\beta_{T}=0$.

This equation has no obvious analytic solution but can be solved numerically. Figure 9 shows a finite-difference solution for the three-spot pattern in Fig. 8, assuming that α=−0.25, $\beta_{L}=\;16$, and $\beta_{T}=0$ (anisotropic permeability and large hydrodynamic dispersion). As before, trajectories become curved. However, spots are no longer elliptical, and their long axes rotate away from principal directions toward streamlines, implying that hydrodynamic dispersion can be differentiated from diffusion anisotropy by alteration and reorientation of spot shapes.

## VII. EXPERIMENTAL RESULTS

Radial concentration was performed with Brilliant Blue FCF, a water-soluble dye. Its diffusion coefficient has been measured in water as $D=5.68\times 10^{-10}\;m^{2}/s$.^66^ Dye was obtained at ≥97% purity (80717, Sigma Aldrich, St. Louis, USA) and dissolved in de-ionized water. Concentration was carried out on 90 mm diameter qualitative circles (1001090, Whatman, Little Chalfont, UK, thickness d=180μm, pore size 11μm), on which the retardation factor of Brilliant Blue is ≈1.^67^

**FIG. 10.**
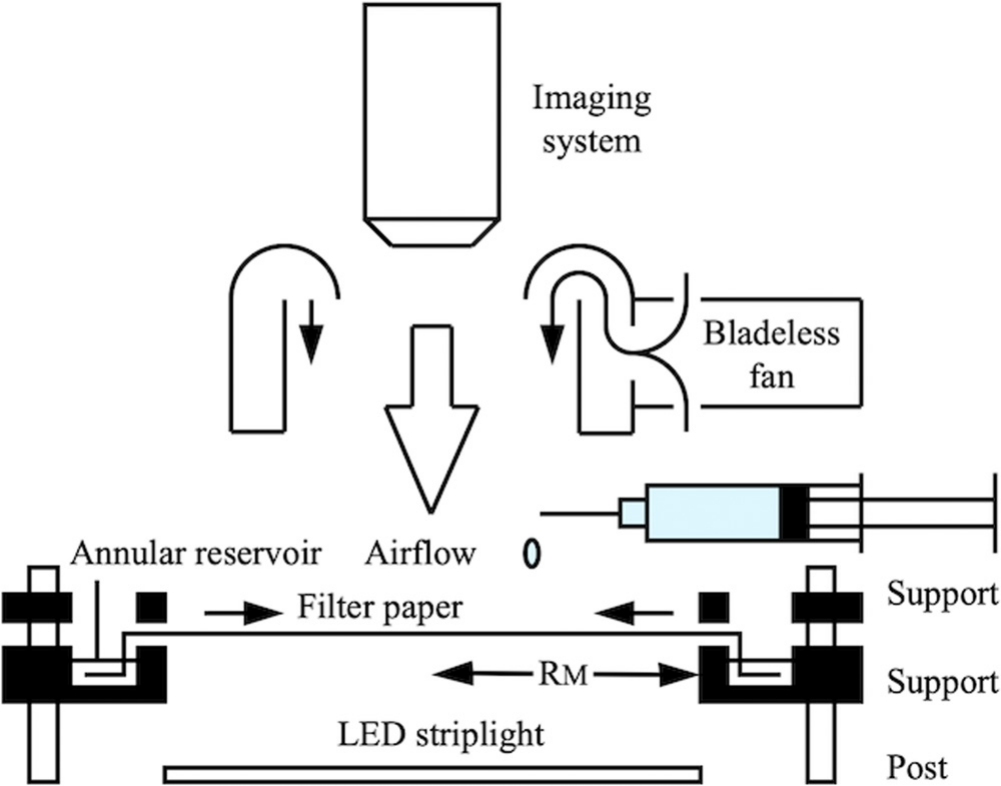
Arrangement for radial concentration.

Experiments were performed in laboratory conditions (20–22.5 °C, 40–65%RH). Despite this variation, results were qualitatively repeatable. Figure 10 shows the arrangement used. The paper was held horizontally between two plastic supports to leave a portion of radius $R_{M}=30\;\mathrm{mm}$ suspended. The lower support contained an annular reservoir linked to the evaporation section, while the upper support acted as a paper-cutting guide. Water drops were dispensed to locate the principal axes. Radial slots were scalpel-cut to allow tabs to be bent into the reservoir, which was filled with de-ionized water, so the paper was fully wetted. Dye spots were then dispensed. Evaporation could be prevented by sealing the plastic jig with transparent lids, performed under ambient conditions, or enhanced by using a bladeless fan (X12, Archuu, Shenzhen, China).

**FIG. 11.**
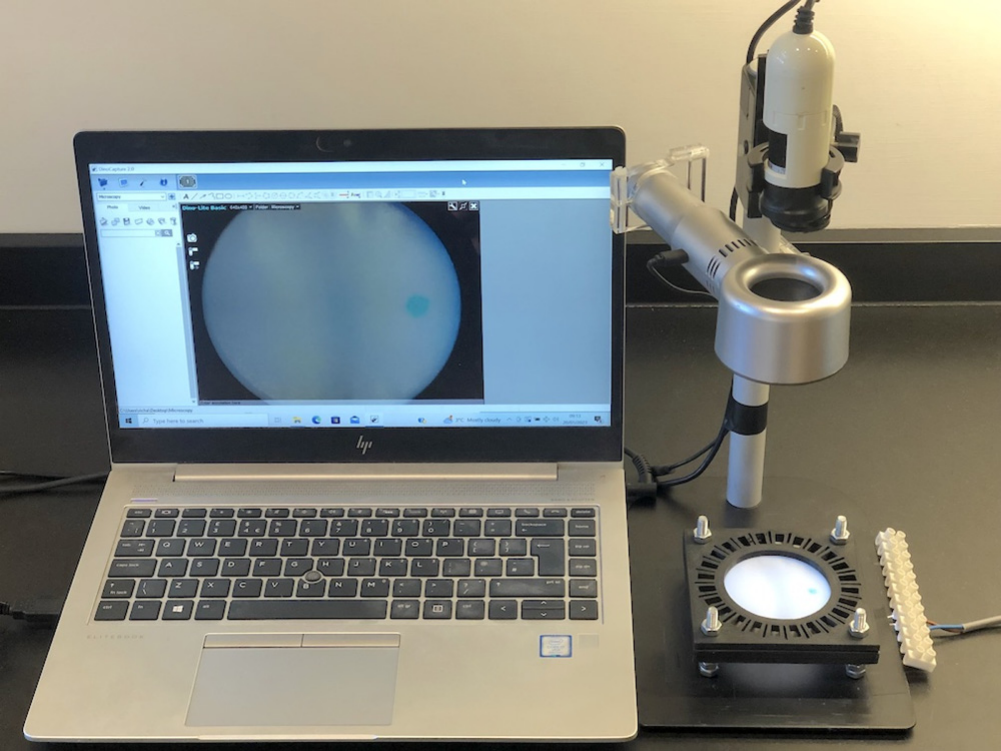
Experimental rig with a microscope and a fan in place.

Diffusion, transport, and concentration were monitored using a Dino-Lite AM2011 Basic USB digital microscope (AnMo Electronics Corp., Taipei, Taiwan), arranged to view through the fan duct, with a white InGaN-on-sapphire LED strip-light (LuckyLight, Shenzhen, China) for illumination. Figure 11 shows the experimental jig with the microscope and fan in place. Spot shapes and trajectories were extracted by post-processing time-lapse photographs to extract transmission profiles from red-channel data and converting to spatial variations in concentration using the Beer–Lambert law. Although the light source is broadband, linearity has been established for the concentration range here.^12^

The diffusion coefficient of Brilliant Blue on wetted papers was found by photographing a single dye spot at different times *T*, with evaporation suppressed. Best-fits between line-scan absorbance data and Gaussian profiles were then obtained in the form

$$A(z)=a\;\exp\left\{-{\left[\left(z-b\right)/c\right]}^{2}\right\}.$$ (51)

**FIG. 12.**
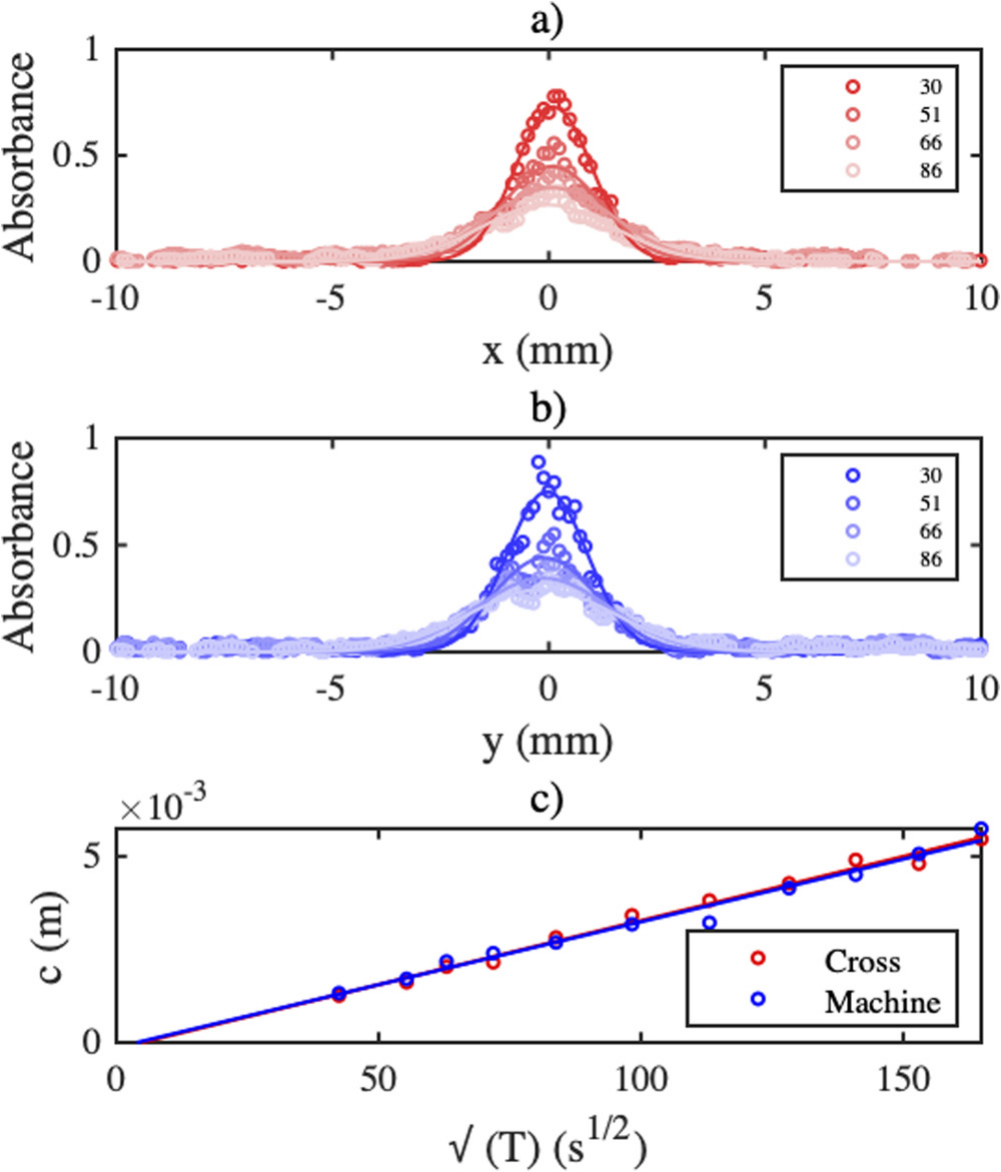
Experimental absorbance profiles at different times *T* in (a) cross and (b) machine directions; (c) variations of the fitting constant *c* with $\sqrt{T}$. Points are data, lines are best fits.

Values of *c* were then extracted, and the diffusion coefficient *D* was estimated by comparison with the solution of Fick's second law,^68^ which implies that $c=\sqrt{4DT}$. Figures 12(a) and 12(b) show example absorbance profiles in the cross and machine directions at the times *T* shown in minutes, compared with Gaussian fits.

Figure 12(c) shows the variations of *c* with $\sqrt{T}$ for the machine and cross directions, compared with straight line fits. Here, the slopes are almost the same, but an average of six data sets yielded $D=3.08\pm 0.11\times 10^{-10}\;m^{2}/s\;$ (machine), and $D=2.31\pm 0.1\times 10^{-10}\;m^{2}/s$ (cross) implying minor diffusion anisotropy. The values are around 45% of the earlier literature value.^66^ This reduction is due to the tortuosity *T* of the paper microstructure, which is, in turn, related to the porosity ε. Many models show an effective diffusion constant of the form $D^{\prime }=D/T^{2}$. One review^69^ yields values of $D^{\prime }/D$ in the range of 0.25–0.4 for ε=0.5, slightly lower than our result. However, the experiments are hard to perform repeatedly. Similarly, diffusion anisotropy is expected from anisotropy in tortuosity.

**FIG. 13.**
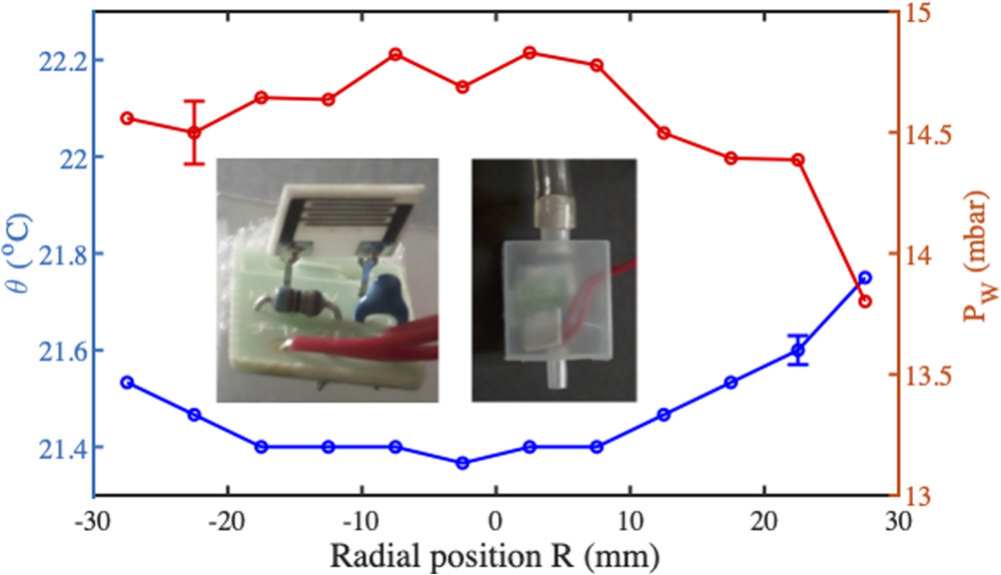
Variations of temperature θ and partial pressure of water $P_{W}$ for ambient evaporation, with representative error bars. Inset: sensor elements and sampling probe.

For ambient evaporation, local temperature and humidity were measured together using a probe constructed from a data logger based on a capacitive humidity sensor and a thermistor (Thermo Recorder TR-72Ui, T&D, Matsumoto City, Japan). After removal from its original housing, the sensor head was mounted in a capsule with a small opening. Air was drawn slowly through the capsule by an axial flow fan, while the probe was translated across the paper at a small (1−2mm) separation from the surface. The inset of Fig. 13 shows the sensor elements and sampling probe. The local partial pressure $P_{W}$ of water was estimated from the saturation vapor pressure $P_{S}$ and the percentage humidity ϕ. $P_{S}$ was estimated in mbar from the Buck equation,^70^

$$P_{S}=(1.0007+3.46\times 10^{-6}P)\times 6.1121e^{\frac{17.502\theta }{240.97+\theta }}.$$ (52)

Here, θ is the dry-bulb temperature in degrees Celsius, and P=1018mbar is the absolute pressure (measured here at a local weather station). $P_{w}$ was then found from humidity measurements, using the relation

$$\phi =100\times P_{W}/P_{S}.$$ (53)

Figure 13 shows variations of θ and $P_{W}$ along a paper diameter, obtained as an average of three data sets. The former falls toward the center of the disk, consistent with evaporative cooling, while the latter rises. Near the center, conditions are approximately constant, confirming the earlier assumption of uniform evaporation. The ambient temperature was 22.6 °C, so the central decrease is 1.2 °C. This value is lower than expected because the thermistor and sampled air both reach thermal equilibrium before each measurement. Although efforts were made to provide insulation, the equilibrium temperature is higher than the inlet air temperature. Furthermore, the extrema measurements are not expected to reach ambient temperature because the probe diameter prevents the inlet being placed directly over the perimeter, and the probe then averages over several mm.

**FIG. 14.**
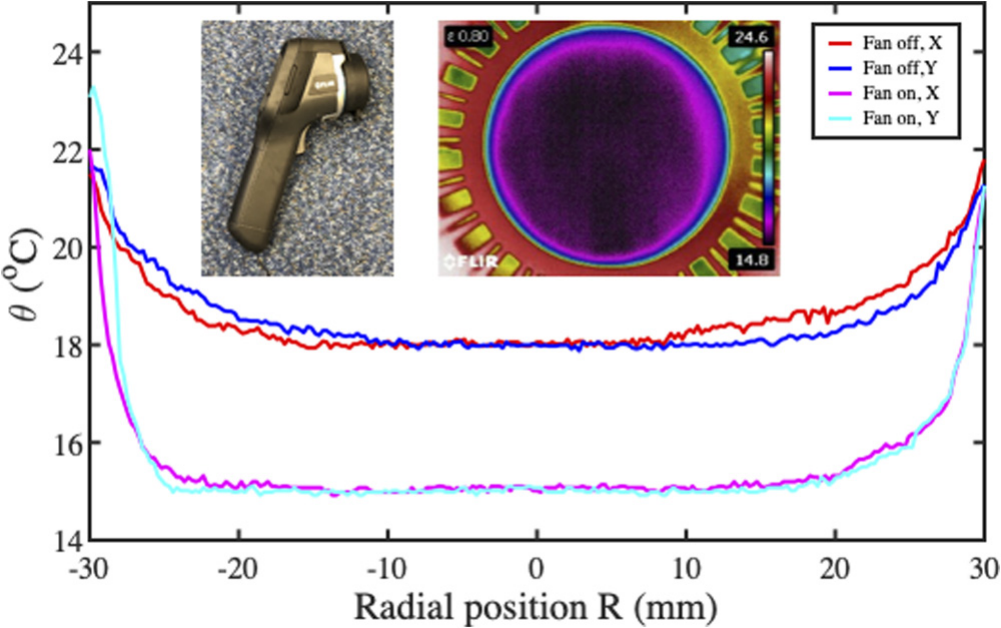
Local variations of temperature, with and without fan assistance. Inset: thermal camera and thermal image of paper with fan-assisted evaporation.

For fan-assisted evaporation, temperature measurements were obtained using a FLIR E60 uncooled microbolometer array (FLIR Systems, Wilsonville, OR). The inset of Fig. 14 shows the camera and the main figure shows temperature profiles along the diameter in perpendicular directions, with and without fan assistance.

In each case, the variation is again bowl shaped, with a near-uniform central section. However, the temperature clearly falls further with fan assistance, and the region of constant temperature increases. The inset also shows a colorized thermal image obtained under these conditions. This contains a large, radially symmetric region of uniform low temperature, further confirming the assumption of uniform evaporation.

**FIG. 15.**
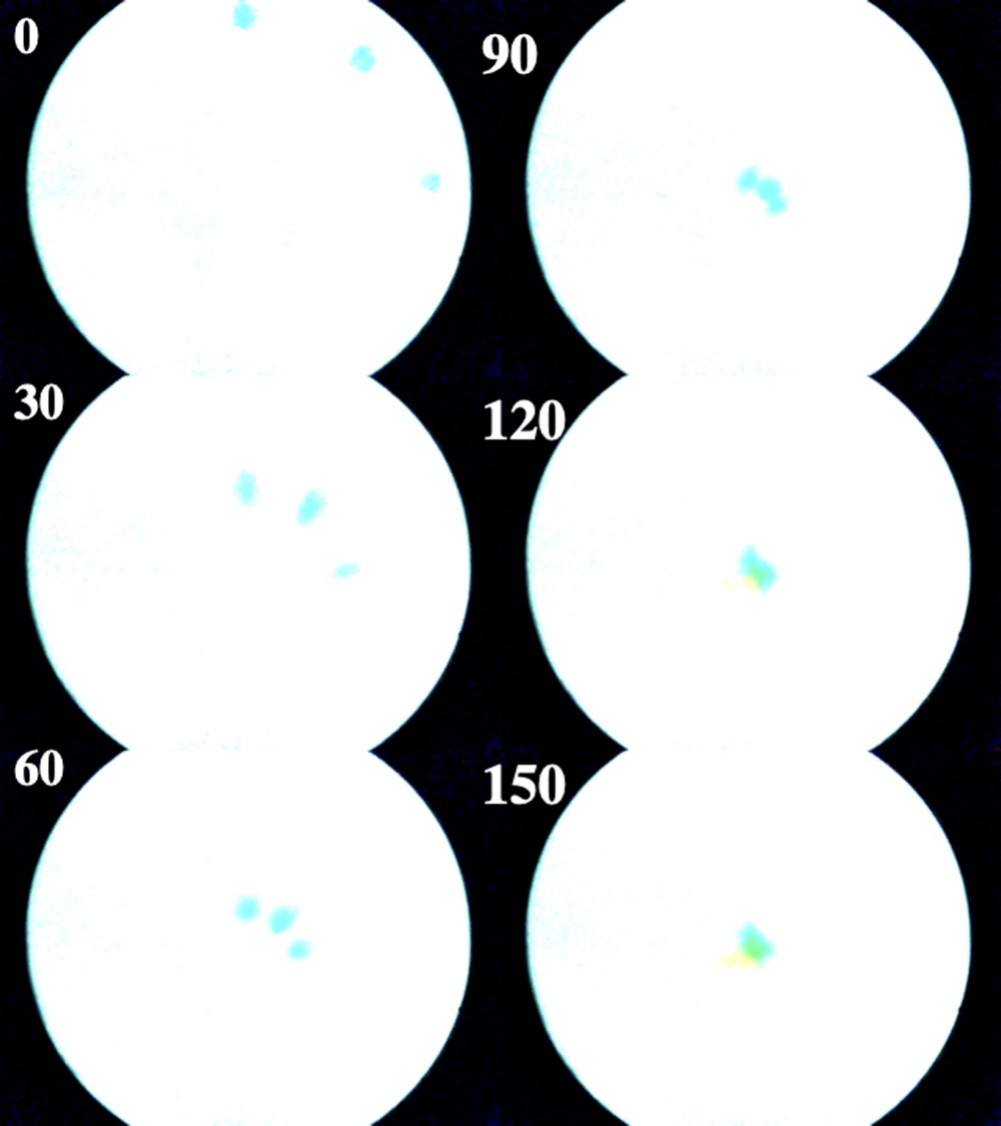
Time-lapse photographs of a three-spot dye distribution, with fan-assisted evaporation.

For ambient evaporation, Péclet numbers were small (∼200) and experiments took many (>10) hours. Large spot sizes complicated spot tracking and degraded the agreement with exponential models. Much larger (>2500) Péclet numbers were obtained with fan assistance, and agreement with theory improved significantly. Figure 15 shows a three-spot distribution at different times (in min) during a typical fan-assisted experiment. The spots travel from the periphery to the center of the disk and coalesce after ∼2.5 h. Radial spot extension indicates minor hydrodynamic dispersion, but its effects disappear near the stagnation point.

**FIG. 16.**
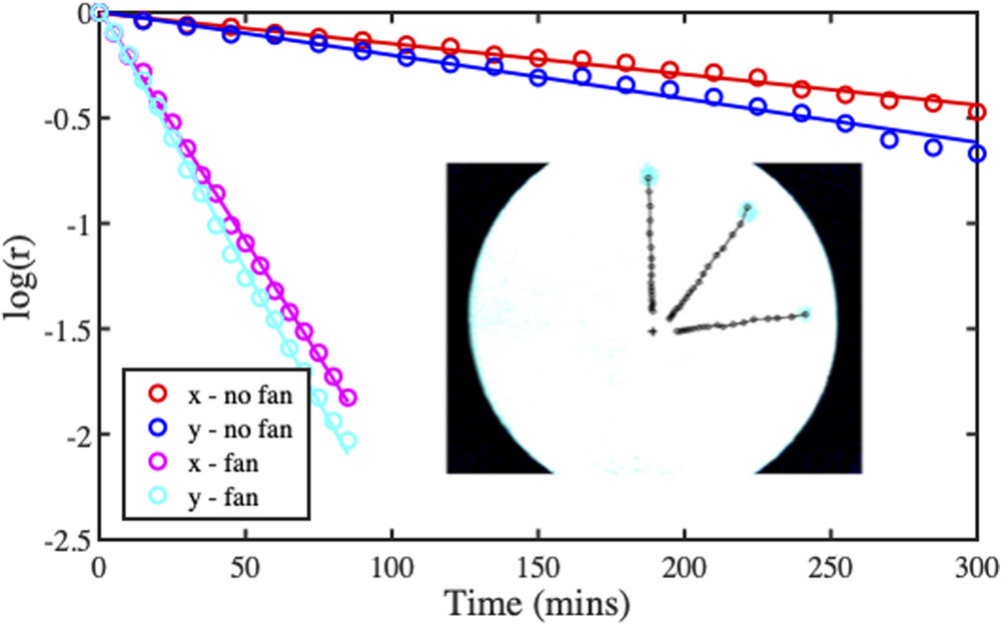
Extracted variations of spot position with time, with and without fan assistance. Points are data, lines are best fits. Inset: spot trajectories with fan assistance.

Figure 16 shows the log-scale time variation of normalized X- and Y-positions for horizontal and vertical trajectories without and with fan assistance, extracted using a peak-finding routine and matched to straight lines with slopes $a_{x}$ and $a_{y}$ using a least squares non-linear algorithm. There is a good match to an exponential variation in each case, and the small difference in slope implies minor anisotropic permeability. The inset shows spot trajectories in the fan-assisted case.

Parameters were extracted as follows. The permeability ratio was estimated as $k_{y}/k_{x}=a_{y}/a_{x}$, the time constant as $\tau_{e}=R_{f}/\left(a_{x}+a_{y}\right)$, the rate of solvent evaporation as $v_{e}=d/\tau_{e}$, and the Péclet number as $Pe=R_{M}^{2}/D\tau_{e}$. Results obtained from Fig. 16 are shown in Table I. There is some variation in permeability ratio, broadly in agreement with published data for paper.^60^ The Péclet number is increased 13-fold by fan-assisted evaporation, rendering timescales realistic for laboratory work.

**TABLE I.** Experimental parameters for ambient and fan-assisted evaporation.

	$k_{y}/k_{x}$	*τ*_*e*_ (s)	*v*_*e*_ (ms^−1^)	*P*_*e*_
Ambient	1.42	1.67 × 10^4^	1.08 × 10^−8^	202
Fan	1.14	1.26 × 10^3^	1.43 × 10^−7^	2687

## VIII. APPLICATIONS

**FIG. 17.**
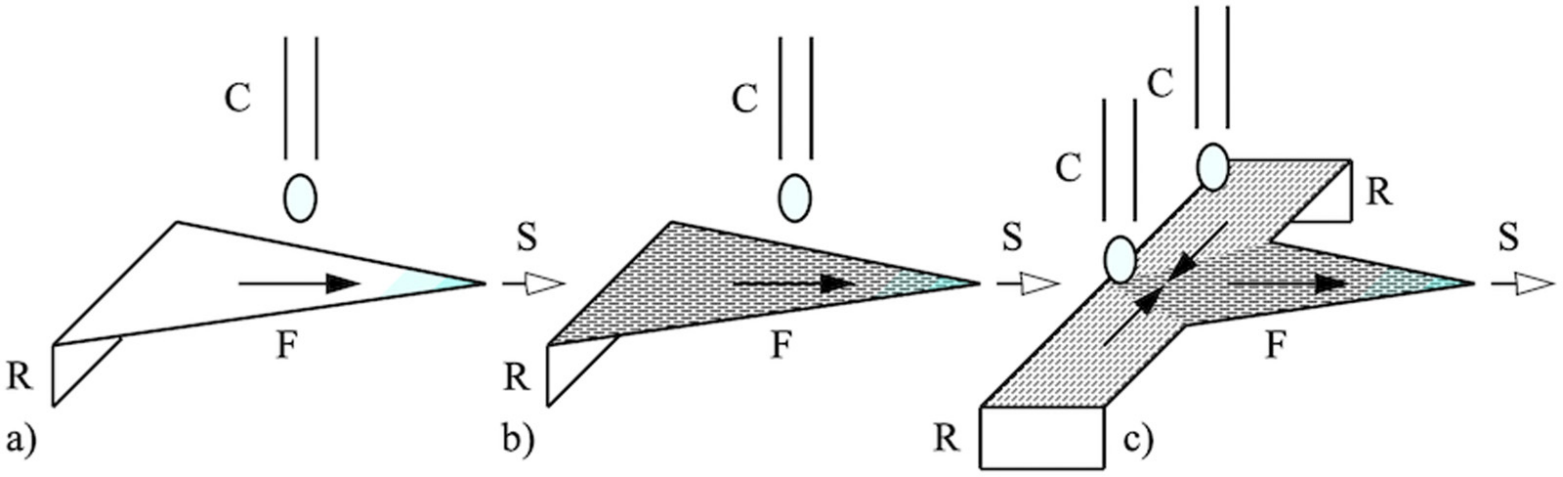
(a) Paper spray source; (b) source with engineered anisotropy; (c) reaction source. R, solvent reservoir; C, capillary dispenser; F, flow; S, electrospray plume.

Paper microfluidics is now highly developed. However, almost all applications are driven by capillary flow; evaporation is considered rarely. Exceptions include paper spray,^71^ where extended solvent supply and desolvation have been adopted,^72,73^ and reaction acceleration on surfaces and in droplets.^74–76^ The previous results show that evaporation pumping can provide concentration as well as transport, especially in radial flow, and can operate over long timescales. Applications are likely to lie in similar areas, as follows:

(1) Concentration: The 2D analysis implies that single droplets will be concentrated at the center of a disk, or, by extension, at the tip of a wedge as shown in Fig. 17(a). The effect must lead to the sensitivity enhancement seen in paper spray mass spectrometry with solvent evaporation, allowing the best use of a limited volume of solute.
(2) Improved paper substrates: The relatively long timescales of evaporation-driven concentration are a limiting factor. However, several techniques are available to manipulate permeability and evaporation by surface patterning. We are exploring laser cutting and have already used the radial-flow apparatus to demonstrate engineered orthotropic anisotropy in permeability. Materials of this type may be used to construct paper spray sources with more effective concentration as shown in Fig. 17(b).
(3) Non-linear effects: The 1D analysis implies that single droplets will be concentrated at the center of a paper strip fed from a reservoir at either end. An analyte and a reagent may then be concentrated simultaneously, so that (for example) an individual concentration factor of 10^2^ leads to an enhancement of 10^4^ in the reaction rate. Arrangements may be combined with paper spray as shown in Fig. 17(c).

Optimizing such applications will require numerical solution of ADEs for more complex geometries and flow fields. However, the initial analysis and experimental results presented here can already allow anticipation of likely effects.

## IX. CONCLUSIONS

Evaporation-driven concentration on porous substrates has been considered in 1D and 2D, and analytic solutions have been found to the ADEs by assuming uniform evaporation and infinite domains. Solutions are constructed from Gaussian kernels following the smoothed particle approximation, which provides an ideal basis for modeling spot trajectories. Provided the Gaussians are well confined, these remain valid over finite domains. The results show that initial distributions always form Gaussian concentration peaks, and that timescales are doubled in the radial geometry. The effect of anisotropic permeability is to modify spot trajectories, while anisotropic diffusion and hydrodynamic dispersion alter spot shapes. Anisotropy can be compensated by operating on the bias or eliminated using isotropic substrates, and hydrodynamic dispersion is unimportant near the stagnation point.

Theoretical predictions are confirmed by radial-flow experiments with water-soluble dyes on filter papers. Further work is required to model and directly measure local evaporation rates, but measurements of temperature, humidity, and spot trajectories all support an assumption of approximately uniform evaporation over most of the paper. Measurements have shown minor anisotropy in both diffusion and permeability. Timescales have been reduced using fan assistance, allowing transport from the perimeter to the center of a 60 mm diameter disk in a few hours. Péclet numbers over 2500 have been achieved, with limited hydrodynamic dispersion. It is likely that faster concentration can be achieved with more powerful airflow, and that the concentration point may be moved using directed air jets.

Radial evaporation-driven concentration may provide a simple method of reconcentrating forensic samples, preconcentrating samples to improve sensitivity in paper spray mass spectrometry or concentrating analytes before reaction with targeted reagents. The advantage over capillary pumping is higher sensitivity and continued operation long after capillary filling has completed. Similarly, the advantage over field-driven pumping is the simplicity of the substrate and lack of any requirement for high voltages. However, environmental control would be needed for fully repeatable results.

Relatively little solvent is required. The necessary volume $V_{SE}$ can be estimated as

$$V_{SE}=\pi R_{M}^{2}v_{e}T.$$ (54)

Ignoring anisotropy in permeability, spot trajectories can be written in terms of $V_{SE}$ as

$$x_{0}=x_{00}\;\exp\left(-V_{SE}R_{f}/2\pi dR_{M}^{2}\right).$$ (55)

To reduce the argument of the exponential to a given value −C, we then require

$$V_{SE}=C\frac{2\pi dR_{M}^{2}}{R_{f}}.$$ (56)

Thus, the exact evaporation rate is unimportant; $V_{SE}$ is dependent only on the substrate dimensions and the solute retardation factor and commensurate with the volume $V_{SF}=\pi dR_{M}^{2}$ needed for filling. Assuming *d*, $R_{M}$, and $R_{f}$ are as previously given, and C=4 (say), we then obtain $V_{SE}\approx 4cc$. However, larger volumes would be required for solutes with lower $R_{f}$. Transport times also increase as $R_{f}$ reduces.

## Data Availability

The data that support the findings of this study are available within the article.
